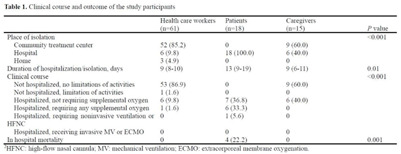# A SARS-CoV-2 outbreak due to vaccine breakthrough in an acute-care hospital

**DOI:** 10.1017/ash.2022.213

**Published:** 2022-05-16

**Authors:** Se Yoon Park, Tae Hyong Kim, Eunjung Lee, Mark Loeb, Yeon Su Jeong, Jin Hwa Kim, Sun Mi Oh, Sojin Cheong, Hyein Park, SoYea Jo

## Abstract

**Background:** The δ (delta) variant has spread rapidly worldwide and has become the predominant strain of SARS-CoV-2. We analyzed an outbreak caused by a vaccine breakthrough infection in a hospital with an active infection control program where 91.9% of healthcare workers were vaccinated. **Methods:** We investigated a SARS-CoV-2 outbreak between September 9 and October 2, 2021, in a referral teaching hospital in Korea. We retrospectively collected data on demographics, vaccination history, transmission, and clinical features of confirmed COVID-19 in patients, healthcare workers, and caregivers. **Results:** During the outbreak, 94 individuals tested positive for SARS-CoV-2 using reverse transcription-polymerase chain reaction (rtPCR) testing. Testing identified infections in 61 health care workers, 18 patients, and 15 caregivers, and 70 (74.5%) of 94 cases were vaccine breakthrough infections. We detected 3 superspreading events: in the hospital staff cafeteria and offices (n = 47 cases, 50%), the 8th floor of the main building (n = 22 cases, 23.4%), and the 7th floor in the maternal and child healthcare center (n = 12 cases, 12.8%). These superspreading events accounted for 81 (86.2%) of 94 transmissions (Fig. 1, 2). The median interval between completion of vaccination and COVID-19 infection was 117 days (range, 18–187). There was no significant difference in the mean Ct value of the RdRp/ORF1ab gene between fully vaccinated individuals (mean 20.87, SD±6.28) and unvaccinated individuals (mean 19.94, SD±5.37, P = .52) at the time of diagnosis. Among healthcare workers and caregivers, only 1 required oxygen supplementation. In contrast, among 18 patients, there were 4 fatal cases (22.2%), 3 of whom were unvaccinated (Table 1). **Conclusions:** Superspreading infection among fully vaccinated individuals occurred in an acute-care hospital while the δ (delta) variant was dominant. Given the potential for severe complications, as this outbreak demonstrated, preventive measures including adequate ventilation should be emphasized to minimize transmission in hospitals.

**Funding:** None

**Disclosures:** None

**Figure:**
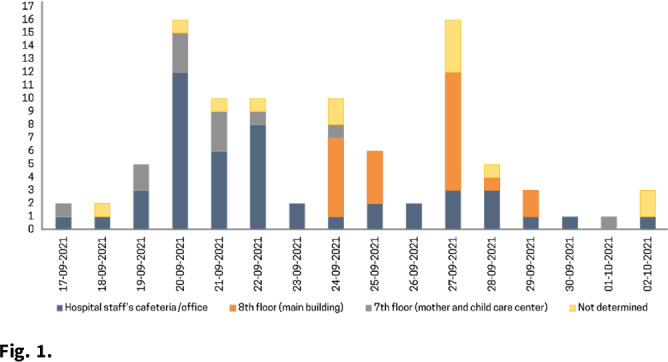

**Figure:**
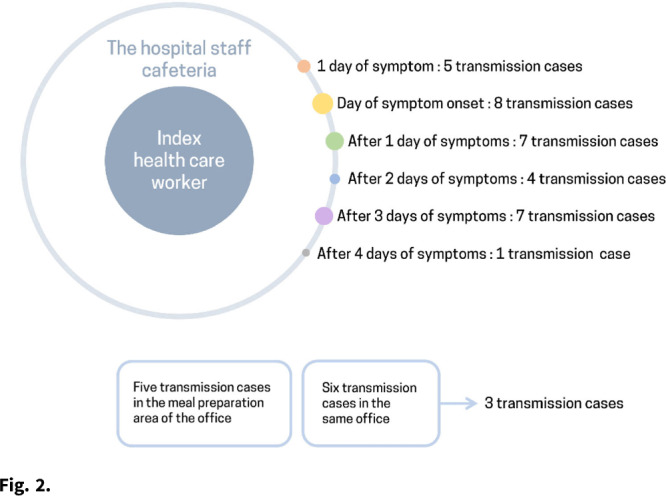

**Table:**